# Performance analysis of 3-D shape measurement algorithm with a short baseline projector-camera system

**DOI:** 10.1186/s40638-014-0001-8

**Published:** 2014-08-28

**Authors:** Jianyang Liu, Youfu Li

**Affiliations:** Department of Mechanical and Biomedical Engineering, City University of Hong Kong, Kowloon, Hong Kong

**Keywords:** 3-D shape measurement, Projector-camera, Short baseline, Binocular stereovision, FPP

## Abstract

A number of works for 3-D shape measurement based on structured light have been well-studied in the last decades. A common way to model the system is to use the binocular stereovision-like model. In this model, the projector is treated as a camera, thus making a projector-camera-based system unified with a well-established traditional binocular stereovision system. After calibrating the projector and camera, a 3-D shape information is obtained by conventional triangulation. However, in such a stereovision-like system, the short baseline problem exists and limits the measurement accuracy. Hence, in this work, we present a new projecting-imaging model based on fringe projection profilometry (FPP). In this model, we first derive a rigorous mathematical relationship that exists between the height of an object’s surface, the phase difference distribution map, and the parameters of the setup. Based on this model, we then study the problem of how the uncertainty of relevant parameters, particularly the baseline’s length, affects the 3-D shape measurement accuracy using our proposed model. We provide an extensive uncertainty analysis on the proposed model through partial derivative analysis, relative error analysis, and sensitivity analysis. Moreover, the Monte Carlo simulation experiment is also conducted which shows that the measurement performance of the projector-camera system has a short baseline.

## Introduction

Noncontact optical measurement methodology has been widely used in many industrial applications, such as industry inspection and 3-D printing manufacturing. Among these mature optical 3-D measurement techniques, the structured light technique has been widely used in recent years due to its good characteristics of high precision, flexibility, and robustness to texture-less object surface reconstruction. Numbers of works have been presented in this issue [1–3]. According to a different model, the methods for obtaining 3-D shape information with a structured light system can be simply divided into two main categories: one common way is using conventional binocular stereo vision or named ‘CSV’ model and the other strategy is adopting fringe projection profilometry (FPP) technique. In the first category, the projector is always treated like a camera. In this model, projector and camera in the system are always required to be pre-calibrated before 3-D shape measurement task. Many camera calibration methods are available to be utilized directly [4,5]. However, for projector calibration, even with the latest accurate calibration methods using active target and phase-shifting technique [17,18], the accuracy of projector calibration can hardly reach as the same level as the camera. One of the intuitive reasons is that the parameters of a projector cannot be calibrated individually without the help of camera. Thus, the error propagation from camera calibration process is unavoidable and the overall system calibration accuracy is limited. The biased calibrated parameters of camera and projector will decrease the measurement accuracy. Particularly, in a short baseline arrangement system, the bias from feature point localization on the image will also be magnified with biased parameters. Therefore, how to accurately calibrate a short baseline arrangement system is more critical than a general configuration system which usually has a much larger baseline. In the second category, the projector is commonly regarded as grating optical device. The height information is obtained from the phase-to-height mapping relationship between phase distribution and geometric parameters of the system. Hence, the projector is not needed to be pre-calibrated anymore. In some presented phase-to-height model, even the camera is also not required to be pre-calibrated [7,8].

In this paper, our method falls into the second category. We propose a generic FPP-based projecting-imaging model and explore the relationship between the phase distribution, height information, and geometric parameters of the system. Based on the proposed model, we then study the problem of how the uncertainty of relevant parameters, the length of baseline in particular, affects the 3-D shape measurement accuracy. In other words, we focus on the performance analysis of the 3-D shape measurement according to our proposed model particularly when the system has a short baseline.

## Background and literature review

### Conventional stereovision model

**Figure 1:**
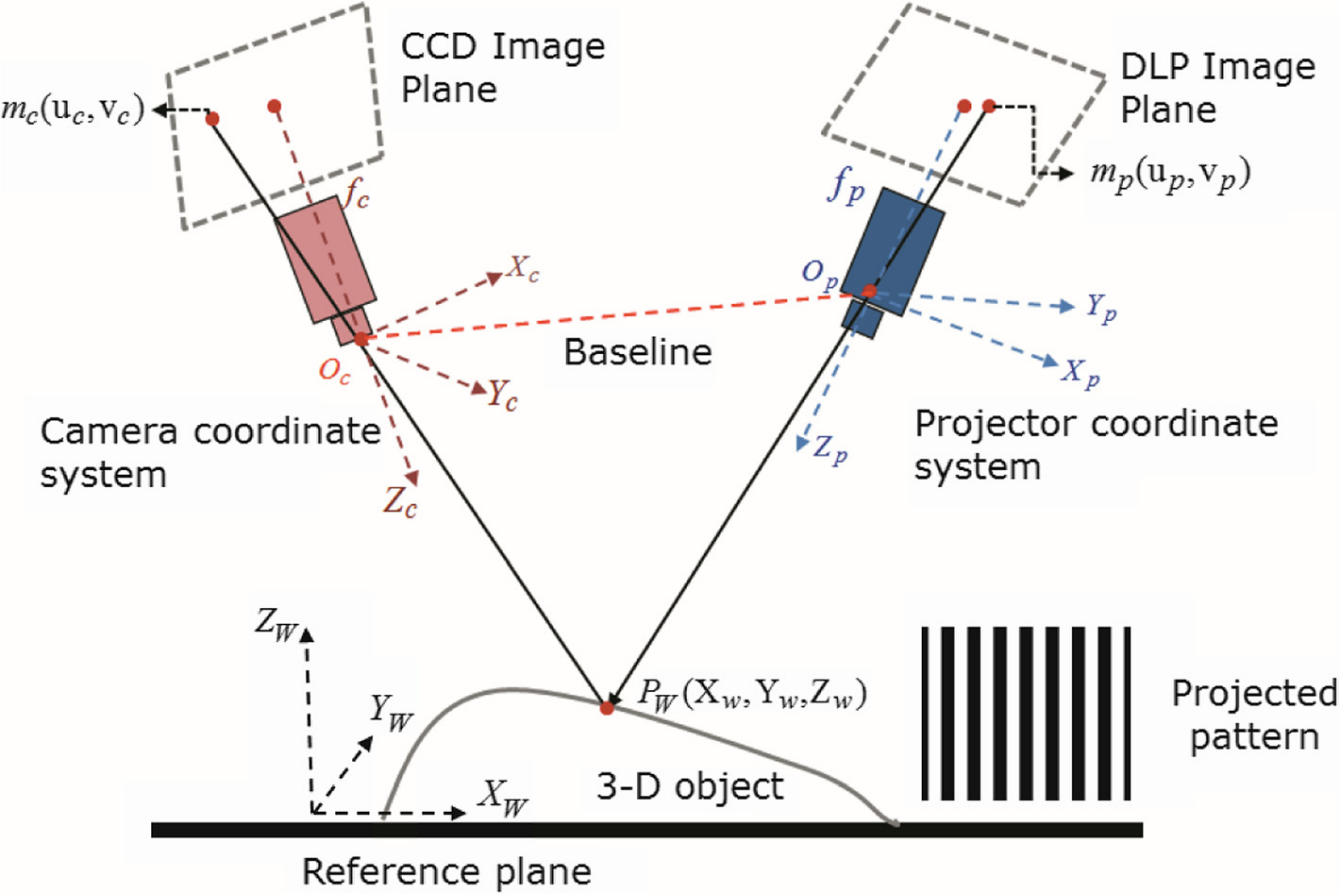
**Schematic diagram of a Pro-Cam structured light system.**

The schematic diagram of a projector-camera (Pro-Cam) structured light system is illustrated in Figure 1. The key problem in the 3-D shape measurement process is how to determine the corresponding relationship between the point on camera’s image plane, projector’s image plane, and the point on the object’s surface. It is worthy noticing that these three points also represent the three vertices of a triangle, respectively. Generally, the structured light pattern projected from a projector plays a role of bridge. Once the relationship between the projector image plane and camera image plane is established, the short baseline arranged 3-D shape measurement system, which is illustrated in Figure 1, can be unified with a classic binocular stereovision system.

Given a point on the camera’s image plane and its corresponding point on the projector’s image plane, the coordinate of its corresponding point on the object’s surface can be determined by conventional triangulation algorithm [16,19]. The relationship between nondistorted point *m*_*u*_(*u*_*c*_, *v*_*c*_) on the camera’s image plane and its corresponding point *P*_*W*_ = [*X*^*W*^, *Y*^*W*^, *Z*^*W*^]^*T*^ on the object’s surface can be described as follows:

1 $$ {s}_c\left[\begin{array}{c}\hfill {u}_c\hfill \\ {}\hfill {v}_c\hfill \\ {}\hfill 1\hfill \end{array}\right]=\left[\begin{array}{ccc}\hfill {f}_{c x}\hfill & \hfill {\lambda}_c\hfill & \hfill {u}_{c0}\hfill \\ {}\hfill 0\hfill & \hfill {f}_{c y}\hfill & \hfill {v}_{c0}\hfill \\ {}\hfill 0\hfill & \hfill 0\hfill & \hfill 1\hfill \end{array}\right]\left[\begin{array}{cccc}\hfill {R}_{11}^c\hfill & \hfill {R}_{12}^c\hfill & \hfill {R}_{13}^c\hfill & \hfill {t}_1^c\hfill \\ {}\hfill {R}_{21}^c\hfill & \hfill {R}_{22}^c\hfill & \hfill {R}_{23}^c\hfill & \hfill {t}_2^c\hfill \\ {}\hfill {R}_{31}^c\hfill & \hfill {R}_{32}^c\hfill & \hfill {R}_{33}^c\hfill & \hfill {t}_3^c\hfill \end{array}\right]\left[\begin{array}{c}\hfill {X}_W\hfill \\ {}\hfill {Y}_W\hfill \\ {}\hfill {Z}_W\hfill \\ {}\hfill 1\hfill \end{array}\right] $$

where (*u*_*c*0_, *v*_*c*0_) is the coordinate of the principle point. *f*_*cx*_ and *f*_*cy*_ are the focal length in pixels of the camera image plane along *u* and *v* axes, *λ*_*c*_ denotes the skewness of the two image axes on the camera’s image plane. Then perspective transformation of camera imaging process can be simplified from Equation 1 and denoted as

2 $$ \left[\begin{array}{c}\hfill {s}_c{u}_2^c\hfill \\ {}\hfill {s}_c{v}_2^c\hfill \\ {}\hfill {s}_c\hfill \end{array}\right]=\left[\begin{array}{cccc}\hfill {A}_{11}\hfill & \hfill {A}_{12}\hfill & \hfill {A}_{13}\hfill & \hfill {A}_{14}\hfill \\ {}\hfill {A}_{21}\hfill & \hfill {A}_{22}\hfill & \hfill {A}_{23}\hfill & \hfill {A}_{24}\hfill \\ {}\hfill {A}_{31}\hfill & \hfill {A}_{32}\hfill & \hfill {A}_{33}\hfill & \hfill {A}_{34}\hfill \end{array}\right]\left[\begin{array}{c}\hfill {X}_W\hfill \\ {}\hfill {Y}_W\hfill \\ {}\hfill {Z}_W\hfill \\ {}\hfill 1\hfill \end{array}\right]. $$

Similar for the projector, using phase-shifting technique [17], we can obtain a similar relationship:

3 $$ \left[\begin{array}{c}\hfill {s}_p{u}_1^p\hfill \\ {}\hfill {s}_p{v}_1^p\hfill \\ {}\hfill {s}_p\hfill \end{array}\right]=\left[\begin{array}{cccc}\hfill {B}_{11}\hfill & \hfill {B}_{12}\hfill & \hfill {B}_{13}\hfill & \hfill {B}_{14}\hfill \\ {}\hfill {B}_{21}\hfill & \hfill {B}_{22}\hfill & \hfill {B}_{23}\hfill & \hfill {B}_{24}\hfill \\ {}\hfill {B}_{31}\hfill & \hfill {B}_{32}\hfill & \hfill {B}_{33}\hfill & \hfill {B}_{34}\hfill \end{array}\right]\left[\begin{array}{c}\hfill {X}_W\hfill \\ {}\hfill {Y}_W\hfill \\ {}\hfill {Z}_W\hfill \\ {}\hfill 1\hfill \end{array}\right]. $$

Arranging the variables, we can get the following relationship from Equations 2 and 3.

4 $$ K\cdot P= Q, $$

where $$ K=\left[\begin{array}{ccc}\hfill {A}_{11}-{A}_{31}{u}_2^c\hfill & \hfill {A}_{12}-{A}_{32}{u}_2^c\hfill & \hfill {A}_{13}-{A}_{33}{u}_2^c\hfill \\ {}\hfill {A}_{21}-{A}_{31}{v}_2^c\hfill & \hfill {A}_{22}-{A}_{32}{v}_2^c\hfill & \hfill {A}_{23}-{A}_{33}{v}_2^c\hfill \\ {}\hfill {B}_{11}-{B}_{31}{u}_1^p\hfill & \hfill {B}_{12}-{B}_{32}{u}_1^p\hfill & \hfill {B}_{13}-{B}_{33}{u}_1^p\hfill \\ {}\hfill {B}_{21}-{B}_{31}{v}_1^p\hfill & \hfill {B}_{22}-{B}_{32}{v}_1^p\hfill & \hfill {B}_{23}-{B}_{33}{v}_1^p\hfill \end{array}\right] $$ is a 4 × 3 matrix, $$ Q=\left[\begin{array}{c}\hfill {A}_{34}{u}_2^c-{A}_{14}\hfill \\ {}\hfill {A}_{34}{v}_2^c-{A}_{24}\hfill \\ {}\hfill {B}_{34}{u}_1^p-{B}_{14}\hfill \\ {}\hfill {B}_{34}{v}_1^p-{B}_{24}\hfill \end{array}\right] $$ is a vector, and *P* = [*X*^*W*^, *Y*^*W*^, *Z*^*W*^]^*T*^. It is worth to note that if we are given the real distorted image point *m*_*d*_, it has to be transformed to the nondistorted point *m*_*u*_ first. However, it is difficult to directly get the analytical inversion from distorted image point to nondistorted image point. In this work, the iterative method [12] is adopted and the iteration relationship is given as follows:

5 $$ \left\{\begin{array}{c}\hfill {m}_u={m}_d-\left({k}_1{r}^2+{k}_2{r}^4\right){m}_u+\left[\begin{array}{c}\hfill 2{k}_3{x}_u{y}_u+{k}_4\left({r}^2+2{x_u}^2\right)\hfill \\ {}\hfill {k}_3\left({r}^2+2{y_u}^2\right)+2{k}_4{x}_u{y}_u\hfill \end{array}\right]\hfill \\ {}\hfill {m}_d={m}_u\hfill \end{array}\right.. $$

Therefore, if we know the real pixel point pair ${m}_d^c\left({u}_d^c,{v}_d^c\right)$ and ${m}_d^p\left({u}_d^p,{v}_d^p\right)$, the remainder point in the triangle which is also the corresponding point in 3-D space *P*_*W*_(*X*_*W*_, *Y*_*W*_, *Z*_*W*_)^*T*^, can be obtained by

6 $$ P={\left({K}^T K\right)}^{-1}{K}^T Q. $$

**Figure 2:**
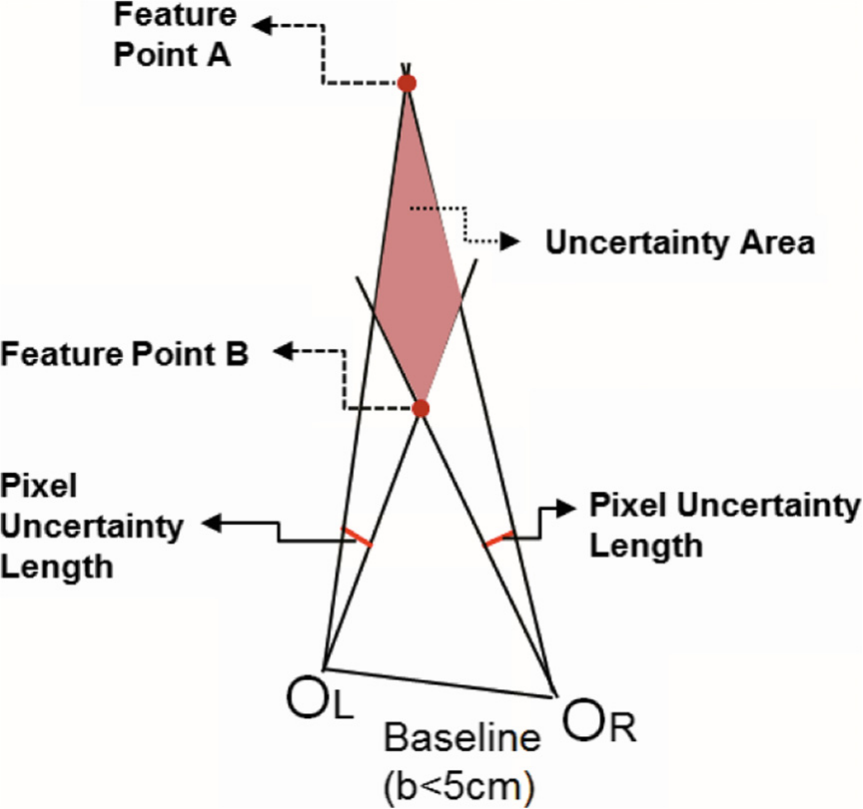
**A brief structure of a short baseline stereovision system setup.**

It is well known that 3-D shape measurement accuracy can be improved through appropriately enlarging the baseline between two optical devices [6,15]. One intuitive reason is that by enlarging the baseline, the ambiguity of the correspondence problem which lies between the pixel on the right camera’s image plane and left camera’s image plane can be alleviated. A brief schematic of a short baseline stereovision system setup is shown in Figure 2. Because the pixel on the image plane has a certain physical size, the feature point *A* and the feature point *B* lie in the same uncertainty area (UA) which is denoted as blue rhombus. All the points lying in this area are corresponding to the same pixel point on the right camera and left camera’s image plane, respectively. In other words, all the points lying in the same UA present the same depth information. Hence, the short baseline problem can be defined, that is, the depth measurement error, which can be denoted as UA shown in Figure 2, will be enlarged in a short baseline system.

Equation 6 shows that an accurate measurement result is depending on the four factors: (1) accurate determination of the pixel point *m*_*cu*_(*u*_*c*_, *v*_*c*_)^*T*^ on camera’s image plane, (2) unbiased camera’s parameters, (3) unbiased projector’s parameters, and (4) accurate determination of the pixel point which corresponds to the pixel point on the projector’s image plane. The first two conditions are easy to be satisfied with available camera calibration algorithm [4,18] and well-developed image processing technique [19,20]. However, the latter two are much more difficult to be achieved. Furthermore, since the projector cannot ‘capture’ like a camera, how to determine the corresponding pixel point on the projector’s image plane is a challenge. If the pixel point on the projector’s image plane is biased, as Figure 2 shows, the measurement error will be enlarged in a short baseline arranged Pro-Cam system.

### FPP-based phase-to-height mapping model

**Figure 3:**
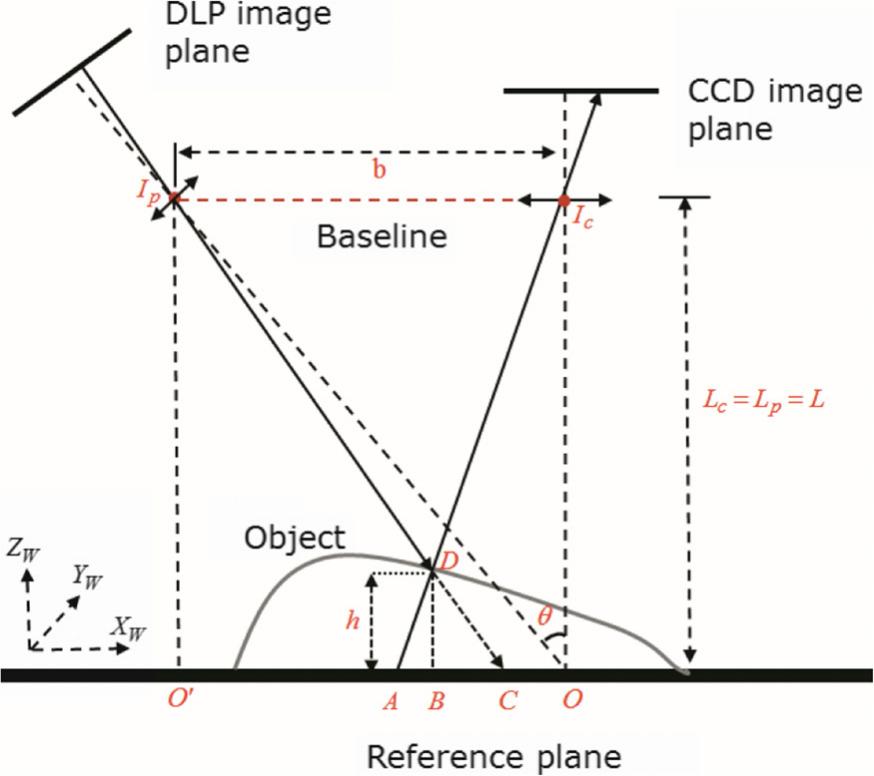
**The basic geometry setup of a Pro-Cam system.**

Fringe projection techniques have been used for 3-D object surface measurement for years because of its flexibility and good performance characteristic. In these techniques, the projector is commonly regarded as grating optical device, and series of fringe patterns (commonly sinusoidal fringe patterns) are projected onto an object’s surface and then captured from other direction by a camera. The captured fringe patterns are deformed with respect to the geometry of the object’s surface. Hence, the intensity distribution of the deformed pattern on the image plane can be retrieved through phase-measuring techniques. One of the classic techniques is Fourier-transform analysis [7]. The other widely used technique is phase-shifting algorithm [2]. Whichever technique is adopted, the critical final step is to create a mapping relationship between the pixel point on the image plane, its corresponding phase, and the height information. One of the basic geometry setup of the measuring system is shown in Figure 3. In the setup, the optical axis *I*_c_*O* of the imaging system camera system is perpendicular to the reference plane. The optical axis *I*_p_*O* of the projection system intersects with the optical axis *I*_c_*O* at point *O* and makes an angle *π*/2 − *θ* with the reference plane. The line joining the two optical centers is the baseline *b*, and the projector and the camera have equal height *L*_p_ = *L*_c_ = *L* with respect to the reference plane. In the work in [8], the phase and height relationship is simply derived using the triangulation method, which is

7 $$ h\left(\mathrm{x},\mathrm{y}\right)=\lambda \left|\Delta {\varphi}_{AD}\right|, $$

8 $$ \lambda = Lp/2\pi b. $$

However, there are two hypotheses assumed in this work. Firstly, the distance between the camera’s optical center and the reference plane is much larger than the height of the object, which is *L* ≫ *h* in general case. The other assumption is that the periodicity of projected fringe pattern, which is denoted as *p* on the reference plane, is evenly distributed along with axis *X*_*w*_. However, in the practical case, the above hypothesis conditions are just two ideal conditions. A thorough analysis in [8] indicates that the periodicity of projected fringe pattern which distributes on the reference plane is in fact a function of the lateral coordinate *x*, the periodicity *p*_0_ of the projecting pattern on the LCD image plane, and the angle *θ* between projector and camera. Hence, in the work in [9], based on the same geometry system setup shown in Figure 3, a more practical expression for phase and height relationship is given as follows:

9 $$ h\left(\mathrm{x},\mathrm{y}\right)= L/\left[\frac{2\pi {L}^2 b \cos \theta}{p_0\left|\Delta {\varphi}_{AD}\right|{\left( L+ x cos\theta sin\theta \right)}^2}-\frac{b \cos \theta \sin \theta}{L+ x \cos \theta \sin \theta}+1\right]. $$

**Figure 4:**
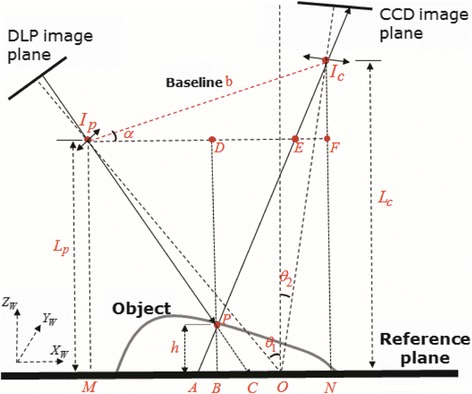
**A generic geometry setup of a Pro-Cam system.**

More accurate results were reported in this work. However, the requirement of parallelity and orthogonality in the work in [8,10] still limited the generality and flexibility in the actual measurement. Therefore, an improved structure of the measurement system is presented by the work in [3]. In this structure, which is shown in Figure 4, the line joining the optical centers of the projector system and camera system is not parallel to the reference plane but makes an angle with the reference plane. In addition, both optical axes are not required to be orthogonal to the reference plane. Comparing with the structure in the basic setup shown in Figure 3, it is more general and closer to practical situation.

After transforming the World Coordinate System to the charge-coupled device (CCD) imaging coordinate system, a final phase-to-height relationship in given as follows [3]:

10 $$ h\left(\mathrm{x},\mathrm{y}\right)=\frac{C_1\left|\Delta {\varphi}_{AD}\right|+{C}_2 u\left|\Delta {\varphi}_{AD}\right|}{1+{C}_3 u+{C}_4\left|\Delta {\varphi}_D\right|+{C}_5\left|\Delta {\varphi}_{AD}\right|+{C}_6 u\left|\Delta {\varphi}_D\right|+{C}_7 u\left|\Delta {\varphi}_{AD}\right|} $$

where coefficients *C*_1_ to *C*_7_ are related to the geometric parameters of the measuring system and the intrinsic and extrinsic parameters of the imaging system. It is worthy noticing that there is another work presented by Du and Wang [1]. In this work, the two optical devices (camera and projector) are arbitrarily arranged; in other words, the geometry structure is also generic and has no special restriction. In other words, it implies that their model fits the case where the baseline between two optical devices could be as small as it could. The phase-to-height mapping relationship was given similarly like in Equation 10, which is described as follows:

11 $$ h\left(\mathrm{x},\mathrm{y}\right)=\frac{C_0+{C}_1{\varphi}_D+\left({C}_2+{C}_3{\varphi}_D\right){I}_D+\left({C}_4+{C}_5{\varphi}_D\right){\mathrm{J}}_D}{D_0+{D}_1{\varphi}_D+\left({D}_2+{D}_3{\varphi}_D\right){I}_D+\left({D}_4+{D}_5{\varphi}_D\right){\mathrm{J}}_D}, $$

where coefficients *C*_0_ to *C*_5_ and *D*_0_ to *D*_5_ are related to the geometric parameters of the measuring system and the intrinsic and extrinsic parameters of the imaging system. As we can see from Equations 10 and 11, in either of the works [1] and [3], the parameters which are physically meaningful (i.e., the length of the baseline) in the presented phase-to-height mapping model are hardly analyzed due to the difficulty of isolating these parameters from the calibrated coefficients. Moreover, they utilized a least-square method to calibrate the coefficients, yet not the related geometric parameters. Therefore, it is necessary to present a practical and analyzable model for convenient analyzing the geometry parameters. In particular, due to the specificity of our proposed short baseline arrangement system, the baseline’s length influence is given the priority to be analyzed. Based on the generic setup in the work [3], we derive a new and different model for accurate phase-to-height mapping determination and parameters analysis.

## Research design and methodology

In the following, a phase-to-height mapping model is presented for parameters’ influence analysis. In this model, a rigorous mathematical relationship that exists between the height of an object’s surface, the phase difference distribution map, and the parameters of the setup is firstly derived. Based on this model, we then study the problem of how the uncertainty of relevant parameters, particularly the baseline’s length, affects the 3-D shape measurement accuracy. The uncertainty analysis on the proposed model including partial derivative analysis, relative error analysis, and sensitivity analysis are performed. Moreover, the Monte Carlo simulation experiment is also conducted.

## Methods

### Our proposed projecting and imaging model

The geometric optical geometry of our setup is shown in Figure 4. *I*_p_ and *I*_c_ are the exit pupil and entrance pupil of the projector and camera, respectively. The optical axes *I*_p_*O* and *I*_c_*O* cross the reference plane at point *O* and make angles *θ*_1_ and *θ*_2_ with *Z*_*W*_ axis (i.e., the normal direction of the reference plane), respectively. The baseline between these two optical centers is *I*_p_*I*_c_ = *b*, which is not parallel to the reference plane. *M* is the perpendicular projection point of *I*_p_ on the reference plane, and the distance between them is *I*_p_*M* = *L*_p_. *N* is the perpendicular projection point of *I*_c_ on the reference plane, and the distance between them is *I*_c_*N* = *L*_c_. Point *A* on the reference plane and point *P* on the object surface correspond to the same image pixel location on the CCD plane. Point *C* on the reference plane and point *P* on the object surface are on the same pixel ray projecting from the projector. We add several dashed lines in the figure as guidelines for analysis. The dashed line *I*_p_*F* is parallel with the reference plane and crosses the extension line of *BP* (*BP* = *h*) at point *D*, which intersects with lines *I*_c_*P* and *I*_c_*N* at points *E* and *F* respectively. In this work, we mainly focus on the measurement performance with respect to the influence of one parameter, which is the baseline *b*. Hence, similarly to the work in [10], we can assume that the fringe patterns formed by the projector are parallel to *Y*_*W*_. From the geometry setup in Figure 4, we can get that the triangle *APB* is similar with the triangle *EI*_c_*F*,

12 $$ AB/ EF= PB/{I}_{\mathrm{c}} F= PB/ b \sin \alpha . $$

Similarly, from the fact that triangle *APB* is similar with triangle *ANI*_c_ and triangle *ACP* is similar with triangle *I*_p_*PE*, we can get

13 $$ AB/ AN= PB/{I}_{\mathrm{c}} N= PB/{L}_{\mathrm{c}}, $$

14 $$ AC/ PB={I}_{\mathrm{p}} E/ PD=\left({I}_{\mathrm{p}} F- EF\right)/ PD=\left({L}_{\mathrm{p}} \tan {\theta}_1+{L}_{\mathrm{c}} \tan {\theta}_2- EF\right)/\left({L}_{\mathrm{p}}- PB\right). $$

Submitting Equation 12 into Equation 14, we can get

15 $$ AC/ PB=\left[{L}_{\mathrm{p}} \tan {\theta}_1+{L}_{\mathrm{c}} \tan {\theta}_2-\left( AB\cdot b \sin \alpha / PB\right)\right]/\left({L}_{\mathrm{p}}- PB\right). $$

Note that *AN* = *AC* + *OC* + *ON* = *AC* + *x* + *L*_c_ tan *θ*_2_, submitting this relationship and Equation 16 into 18, we obtain

16 $$ PB={L}_p{L}_c AC/\left[ AC{L}_c+{L}_p{L}_c \tan {\theta}_1+{L}_c^2 \tan {\theta}_2-\left( AC+ x+{L}_c \tan {\theta}_2\right) b \sin \alpha \right] $$

where *p* denotes the periodicity of the fringe patterns on the reference plane under divergent illumination. According to the work in [3], we can get

17 $$ AC= p\cdot \left|{\varphi}_C-{\varphi}_A\right|/2\pi = p\cdot \left|\Delta {\varphi}_{PA}\right|/2\pi $$

Submitting Equation 17 into Equation 16, we can get the final relationship between the phase distribution *φ*(*x*, *y*) and the height information *h*(*x*, *y*), which is expressed as

18 $$ \begin{array}{l} h\left(\mathrm{x},\mathrm{y}\right)=\left({L}_{\mathrm{p}}{L}_{\mathrm{c}} p\left|\Delta {\varphi}_{PA}\left( x, y\right)\right|\right)/[\left( p- pb \sin \alpha \right)\left|\Delta {\varphi}_{PA}\left( x, y\right)\right|-2\pi bx \sin \alpha \\ {}\kern7.5em +2\pi {L}_{\mathrm{p}}{L}_{\mathrm{c}} \tan {\theta}_1+2\pi {L}_{\mathrm{c}}^2 \tan {\theta}_2-2\pi {L}_{\mathrm{c}} \tan {\theta}_2 b \sin \alpha ].\end{array} $$

It can also be written in a concise form as

19 $$ h\left( x, y\right)={c}_1\left|\Delta {\varphi}_{PA}\left( x, y\right)\right|/\left[{c}_2\left|\Delta {\varphi}_{PA}\left( x, y\right)\right|+{c}_3 x+{c}_4\right], $$

where parameters *c*_1_, *c*_2_, *c*_3_, *c*_4_ are related with geometric parameters *L*_p_, *L*_c_, *p*, *b*, *α*, *θ*_1_, *θ*_2_ and can be denoted as

20 $$ \left\{\begin{array}{c}\hfill {c}_1={L}_{\mathrm{p}}{L}_{\mathrm{c}} p,{c}_2= p- pb \sin \alpha \hfill \\ {}\hfill {c}_3=-2\pi b \sin \alpha, {c}_4=2\pi {L}_{\mathrm{p}}{L}_{\mathrm{c}} \tan {\theta}_1+2\pi {L}_{\mathrm{c}}^2 \tan {\theta}_2-2\pi {L}_{\mathrm{c}} \tan {\theta}_2 b \sin \alpha \hfill \end{array}\right.. $$

### Performance analysis

#### Influence of the length of baseline *b*

The geometric parameters of the system setup include the angle between the optical axis of the projector and the camera, the distance between optical center of camera system and reference plane, the focal length of camera system and the periodicity of projected fringe patterns, etc. In this paper, we regard the baseline’s length as the priority factor and focus on the length of baseline’s influence on the final measurement result. From Equation 19, we can transform it into another form, which takes the baseline *b* as an input variable and is expressed as follows:

21 $$ h\left( x, y\right)={K}_1\left( x, y\right)/\left({K}_2\left( x, y\right)-{K}_3\left( x, y\right)\cdot b\right) $$

where $$ \left\{\begin{array}{c}\hfill {K}_1\left( x, y\right)={L}_{\mathrm{p}}{L}_{\mathrm{c}} p\left|\Delta {\varphi}_{PA}\left( x, y\right)\right|\hfill \\ {}\hfill {K}_2\left( x, y\right)= p\left|\Delta {\varphi}_{PA}\left( x, y\right)\right|+2\pi {L}_{\mathrm{p}}{L}_{\mathrm{c}} \tan {\theta}_1+2\pi {L}_{\mathrm{c}}^2 \tan {\theta}_2\hfill \\ {}\hfill {K}_3\left( x, y\right)= p \sin \alpha \left|\Delta {\varphi}_{PA}\left( x, y\right)\right|+2\pi x \sin \alpha +2\pi {L}_{\mathrm{c}} \tan {\theta}_2 \sin \alpha .\hfill \end{array}\right. $$

From Equation 19, we get the relationship between the phase difference and the height information:

22 $$ \left|\Delta {\varphi}_{PA}\left( x, y\right)\right|=\frac{h\left(\mathrm{x},\mathrm{y}\right)\left({c}_3 x+{c}_4\right)}{h{ c}_2-{c}_1}. $$

Similar to the derivative method in the work in [10], the partial derivative of Equation 23 with respect to the baseline *b* is calculated, and Equations 18 and 19 are submitted into the result. We get

23 $$ \partial h\left(\mathrm{x},\mathrm{y}\right)/\partial b=\left({Q}_1{h}^2\left( x, y\right)-{Q}_2{h}^2\left( x, y\right) b-{Q}_3 h\left( x, y\right)\right)/\left({Q}_4-{Q}_5 b\right) $$

where $$ \left\{\begin{array}{l}{Q}_1=2\pi p \sin \alpha \left({L}_{\mathrm{p}}{L}_{\mathrm{c}} \tan {\theta}_1+{L}_{\mathrm{c}}^2 \tan {\theta}_2+ x- xb \sin \alpha +{L}_{\mathrm{c}} \tan {\theta}_2-{L}_{\mathrm{c}} \tan {\theta}_2 b \sin \alpha \right)\\ {}{Q}_2=2\pi p{ \sin}^2\alpha \left({L}_{\mathrm{c}} \tan {\theta}_2+ x b\right)\\ {}{Q}_3=2\pi p{L}_{\mathrm{p}}{L}_{\mathrm{c}}\left( x \sin \alpha +{L}_{\mathrm{c}} \tan {\theta}_2 \sin \alpha \right)\\ {}{Q}_4=2\pi p{L}_{\mathrm{p}}{L_{\mathrm{c}}}^2\left({L}_{\mathrm{p}} \tan {\theta}_1+{L}_{\mathrm{c}} \tan {\theta}_2\right)\\ {}{Q}_5=2\pi p{L}_{\mathrm{p}}{L}_{\mathrm{c}}\left({L}_{\mathrm{c}} \tan {\theta}_2 \sin \alpha + x \sin \alpha \right).\end{array}\right. $$

**Figure 5:**
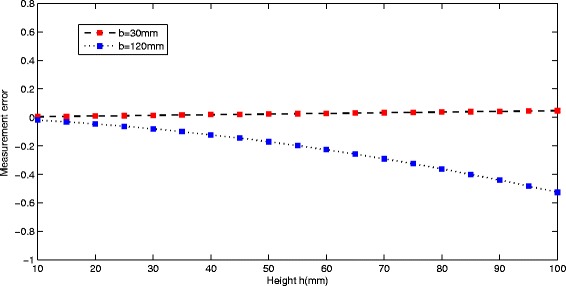
**Plot of the measurement errors** ***∂*** ***h*** **/** ***∂*** ***b*** **versus the height with respect to the different length of baseline.**

Equation 23 shows that the height error ∂*h*(*x*, *y*)/∂*b* is a function of the parameters *Q*_1_, *Q*_2_, *Q*_3_, *Q*_4_, *Q*_5_, *b*, *h*. The dependence of ∂*h*(*x*, *y*)/∂*b* on *h* is shown in Figure 5 with respect to the variation of other parameters *Q*_1_, *Q*_2_, *Q*_3_, *Q*_4_, *Q*_5_. The red curve and blue curve in Figure 5 indicate the lengths of baseline *b* = 30 mm and *b* = 120 mm, respectively. A real experimental system setup consists of a pico-projector (Optoma PK301; Optoma USA, Fremont, CA, USA) and a mini-camera (Point Grey FL3-U3-13S2M-CS; Point Grey Research KK, Chiyoda-ku, Tokyo, Japan**)** with a 6-mm focal length. Hence, the parameters variation are in the following ranges: *L*_p_ from 390 to 420 mm, *L*_c_ from 400 to 450 mm, *p* from 10 to 20 mm, *θ*_1_ from 0° to 15°, *θ*_2_ from 0° to 10°, *α* from 0° to 30°, *x* from −150 to 150 mm.

It is important to note that the parameters in the following analysis are also falling into this range. The results shown in Figure 5 indicate that in the two cases (the baseline *b* = 30 mm and the baseline *b* = 120 mm), the measurement error becomes larger as the height of target object increases. However, in the red curve which indicates the shorter baseline case (*b* = 30 mm), the measurement error is smaller (less than 0.1); meanwhile, the relationship between ∂*h*(*x*, *y*)/∂*b* and *h* is almost linear. On the other hand, when the baseline changes to a larger value (*b* = 120 mm), the relationship between the measurement error ∂*h*(*x*, *y*)/∂*b* and *h* is nonlinear and as the height of the object increases the measurement error increases faster than the shorter baseline (*b* = 30 mm). In particular, when the height of target object equals to 100 mm, the maximum value of the measurement error is larger than 0.5.

### Relative measurement error analysis

A relative measurement error analysis is also conducted with respect to other parameters *K*_1_, *K*_2_, *K*_3_ while the baseline is assumed fixed. Suppose there are small errors *δK*_1_, *δK*_2_, *δK*_3_ existing in the parameters *K*_1_, *K*_2_, *K*_3_, respectively. The relative measurement error of height *δh*/*h* can be expressed by the following generic approximation:

24 $$ \delta h/ h=\sqrt{{\left(\frac{\partial h}{\partial {K}_1}\delta {K}_1\right)}^2+{\left(\frac{\partial h}{\partial {K}_2}\delta {K}_2\right)}^2+{\left(\frac{\partial h}{\partial {K}_3}\delta {K}_3\right)}^2}/ h $$

Submitting Equation 21 into Equation 24, we can obtain

25 $$ \delta h/ h=\sqrt{\delta {K_1}^2+{h}^2\delta {K_2}^2+{b}^2{h}^2\delta {K_3}^2}/{K}_1. $$

Equation 25 indicates that the relative measurement error *δh*/*h* is a function of the length of baseline *b*, the parameters *K*_1_, *K*_2_, *K*_3_, and their variation *δK*_1_, *δK*_2_, *δK*_3_.

**Figure 6:**
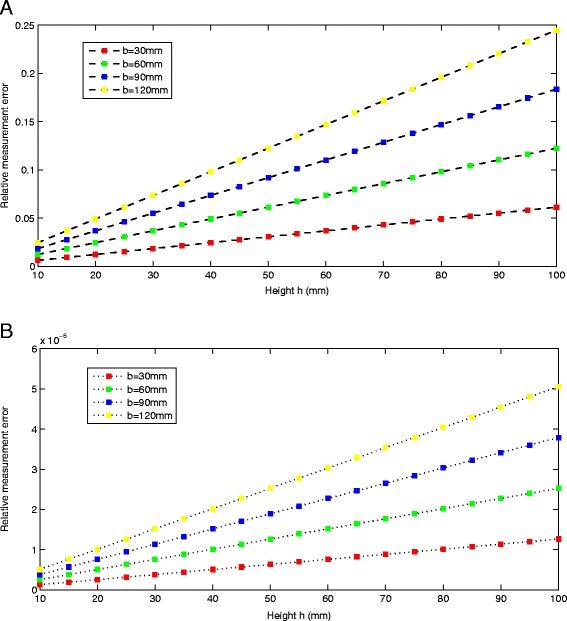
**Plot of the relative measurement error** ***δh*** **/** ***h*** **versus the baseline** ***b.*** **(A)** For parameters *K*_1_,*K*_2_,*K*_3_ go to the minimum value. **(B)** For parameters *K*_1_,*K*_2_,*K*_3_ go to the maximum value.

The results are shown in Figure 6. Without loss of generality, we set the variation of parameters *δK*_1_, *δK*_2_, *δK*_3_ = 0.01. The results tell us that when the height of the target object increases to 100 mm, the yellow curve which represents the largest baseline configuration system (*b* = 120 mm) yields the biggest relative measurement error, which is 25%, 0.0005% for minimizing and maximizing the parameters *K*_1_, *K*_2_, *K*_3_ respectively. Meanwhile, the red curve (*b* = 30 mm) presents the smallest relative measurement error, which is 5%, 0.0001% for minimizing and maximizing the parameters *K*_1_, *K*_2_, *K*_3_ respectively. Hence, we can get the same conclusion that if the length of the baseline increases, the relative error of the measured height becomes bigger, when the same errors *δK*_1_, *δK*_2_, *δK*_3_ are introduced into the system.

### Sensitivity analysis

Furthermore, we take a sensitivity analysis with respect to the baseline *b*. In the following part, *b*_e_ represents the estimates of *b* and Δ*b*/*b* = (*b*_e_ − *b*)/*b* indicates the relative discrepancy with respect to the nominal values. The error ∆*h* can be expressed as the difference between the depth values calculated by substituting the two values *b*_e_ and *b* into Equation 21:

26 $$ \Delta h=\frac{K_1}{K_2-{K}_3{b}_{\mathrm{e}}}-\frac{K_1}{K_2-{K}_3 b}. $$

When Equations 22 and 26 are combined, relative error ∆*h*/*h* results:

27 $$ \Delta h/ h=\frac{K_1}{K_2-{K}_3{b}_{\mathrm{e}}}/\frac{K_1}{K_2-{K}_3 b}-1=-\frac{\varDelta b}{b}\frac{1}{1+\frac{\varDelta b}{b}-\frac{K_2/{K}_3}{b}} $$

**Figure 7:**
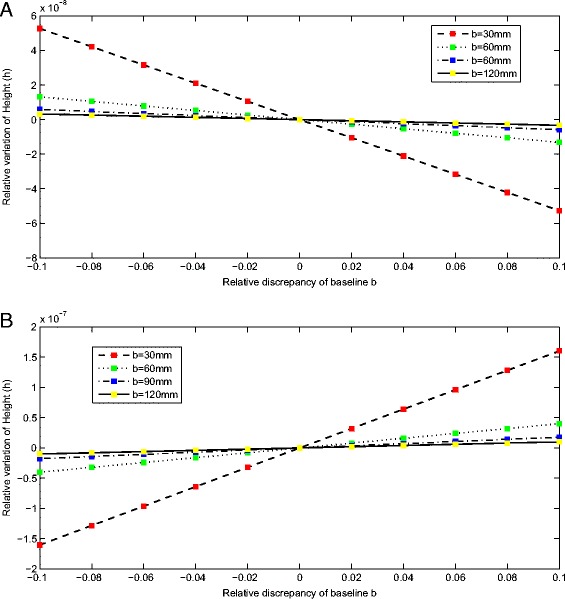
**Plot of relative variation of height ∆** ***h/h*** **as a function of relative discrepancy of baseline ∆** ***b/b*** **. (A)** With respect to the minimum value of parameters *K*_2_ *K*_3_. **(B)** With respect to the maximum value of parameters *K*_2_ *K*_3_.

Equation 27 expresses ∆*h*/*h* as a hyperbolic function of ∆*b*/*b*, but for small values of ∆*b*/*b*, the function is almost linear, as shown in Figure 7. The yellow curve which represents the largest baseline configuration system (*b* = 120 mm) yields the smallest relative variation of height with respect to the same relative discrepancy of baseline, while the red curve which represents the shortest baseline arrangement system (*b* = 30 *mm*) presents the biggest relative variation of height. This means that for a system with a shorter baseline, the proposed model is more sensitive to the small variation of other parameters. In other words, we can get the conclusion that the larger the baseline, the less sensitive is the system with respect to the same bias in the calibrated parameters. This conclusion is the same as presented in the discussion part of the work [11].

## Results and discussion

**Figure 8:**
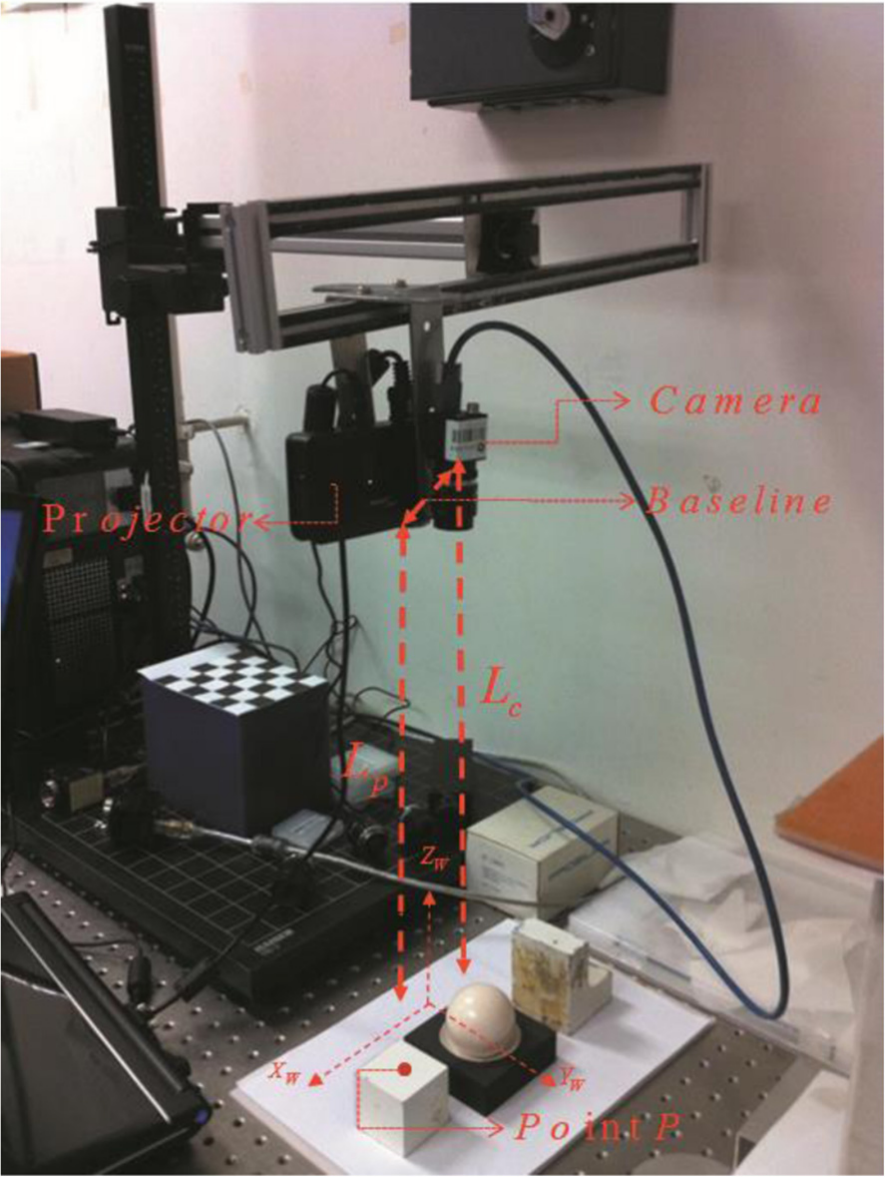
**The experiment setup.**

A measurement error analysis with respect to the variation of baseline has been performed using partial derivative analysis, relative measurement error analysis, and sensitivity analysis. These analysis methods have been limited to parameters *L*_*p*_, *L*_*c*_, *θ*_1_, *θ*_2_, *α*, *p*, *x* and can be accurately calibrated. Parameters *p*, *x* can be easily evaluated with small uncertainty during the measurement by exploitation of the scale factor from pixels to millimeters in the reference frame. On the contrary, accurate determination of parameters *L*_*p*_, *L*_*c*_, *θ*_1_, *θ*_2_, *α* is hard due to the difficulty of precisely measuring the position of the pupils of the projector and of the camera. Meanwhile, it is also hard to determine the accurate relative orientation of the DMD image plane in a DLP-based projector. Hence, in order to eliminate the effect of other unknown uncertainty factors introduced into the analysis process, one way of doing the uncertainty propagation estimation for nonlinear systems is using Monte Carlo analysis [13,14]. The real experimental system setup (shown in Figure 8) consists of a pico-projector (Optoma PK301) and a mini-camera (Point Grey FL3-U3-13S2M-CS) with a 6-mm focal length. Hence, the variation of the parameters can be defined the same as in the previous part.

We present a global sensitivity analysis that permits the evaluation of the uncertainty distribution from other input parameters (i.e., *L*_*p*_, *L*_*c*_, *θ*_1_, *θ*_2_, *α*) to the output (the height information) with respect to the different baseline lengths. In this method, we use four objects with different heights for the experiment. After that, in order to obtain the distribution map of height value, we change the length of baseline and its relevant parameters (i.e., *θ*_1_, *θ*_2_, *α*), but keep the other parameters unchanged. It is worth noticing that the unchanged parameters in the evaluating process are randomly selected from the given range.

**Figure 9:**
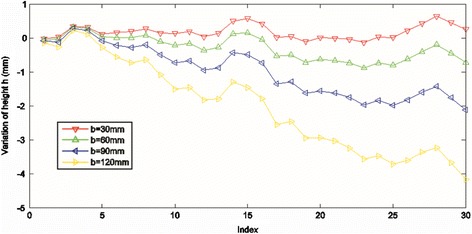
**Plot the variation** **of measured height with respect to the different baseline lengths.**

Figure 9 illustrates the measured height variation (comparing with selected ground truth value) which is corresponding to the random variation of other parameters when the baseline changes from 30 to 120 mm. The initial parameters are randomly selected in the pre-measured range for these four tests. We conduct each set of running for 30 times; in each set, we get the height information from the same sampled point on the object’s surface (i.e., *x* = 188.63 mm). As we can see from the figure, the biggest variations of height are 0.6404, 0.8835, 2.1207, and 4.1729 mm for given baselines of 30, 60, 90, and 120 mm, respectively. Moreover, at each experiment set, when the baseline changes are bigger, the variation of height also increases.

## Conclusions

In this paper, 3-D shape measurement error analysis is performed based on a short baseline Pro-Cam system. The first model is based on conventional stereovision technique. Through analysis, we obtain that as the baseline becomes shorter, the two main factors, which are the inherent biased parameters of the projector and unavoidable biased pixel point localization on the projector’s image plane, have more uncertainty. Therefore, the measurement accuracy is further destroyed. In the second one, we propose a FPP technique-based projecting-imaging model. After deriving a new phase-to-height mapping relationship, measurement error which mainly refers to the height error is analyzed with respect to the length of baseline through partial derivative analysis, relative measurement error analysis, and sensitivity analysis. From the analysis result, we conclude that the smaller the baseline, the more sensitive the system is and the relative measurement error is smaller when if the same biases are introduced in the calibrated parameters. The Monte Carlo simulation experimental results also demonstrate the same measurement result using the proposed model under the short baseline configuration.
